# A New Cryptic *Lactura* from Texas (Lepidoptera, Zygaenoidea, Lacturidae)

**DOI:** 10.3897/zookeys.711.17766

**Published:** 2017-10-23

**Authors:** Tanner Matson, David L. Wagner

**Affiliations:** 1 Department of Ecology and Evolutionary Biology, University of Connecticut, Storrs, Connecticut 06269-3043, USA

**Keywords:** Sapotaceae, gum bully, *Sideroxylon*, DNA barcodes, CO1, *Sideroxylon celastrinum*, tropical burnet moth

## Abstract

A new species of *Lactura* is described from Texas: *Lactura rubritegula* **sp. n.** Identity of the new species can be reliably determined by both larval and adult characters, CO1 haplotypes, and its late-spring period of flight activity. Male genitalic features overlap with those of *L. basistriga* (Barnes & McDunnough, 1913), whereas female structures differ markedly between the pair. The new *Sideroxylon*-feeding species, rare in collections, is found principally in limestone areas in the vicinity San Antonio, Texas, westward through the southern Hill Country. We illustrate the adult and larval stages and male and female genitalia, review available DNA barcode data that support the recognition of the new *Lactura*, and briefly characterize its life history.

## Introduction

The Lacturidae (Tropical Burnet Moths) are a family of zygaenoid lepidopterans that inhabit tropical and subtropical regions of the world. Lacturid adults of North and Central America generally have white to gray forewings marked with antemedial and postmedial rows of red or black spots, and uniformly pink-red hindwings. Species delimitation in the New World has been hampered because this general description applies equally well to nearly all of the ca. 17 North and Central American species (Heppner and Duckworth 1983, Heppner 1984). And to further complicate taxonomic efforts, species such as *L. subfervens* (Walker, 1854) are exceedingly variable in color pattern with forms that overlap those of other members of the genus, including the new species described below.

The most recent checklist of Lepidoptera for the United States and Canada recognizes six species of *Lactura* (Heppner and Duckworth 1983). However, much confusion surrounds the taxonomic identity and validity of some of these species, and especially, the way these names have been applied in institutional collections, literature, genetic databases, and on-line identification resources. The chronic misidentification and misapplication of names within this genus, and the inadequate knowledge of the associated life histories, have made it heretofore difficult to recognize the new North American species described in this work, despite it having salient external features that allow identification of both its adult and larval stages. CO1 barcode data from authoritatively identified adults and larval collections helped us to unmask the existing tangle of misidentifications in collections and internet resources and recognize the new taxon.

Here we describe a new species of *Lactura* from the limestone areas and riparian corridors of south-central Texas’s Hill Country. The new taxon is believed to be a specialist on *Sideroxylon lanuginosum* (Sapotaceae), a host it shares with both *L. pupula* (Hübner, [1831]) and *L. subfervens* at the type locality. We describe and illustrate the larval and adult stages of the new species, illustrate the male and female genitalia, and provide a brief account of its biology and distribution.

## Methods

Adults were obtained by light trapping with UV and mercury-vapor lights. Larvae (of all US species) were collected from *Sideroxylon* (Sapotaceae). Preserved larval specimens were reared from ova deposited by gravid females collected at light traps. Most of the type specimens were collected by Delmar Cain from the type locality in Boerne, Kendall Co., Texas. Additional paratypes were collected by Ed Knudson (Uvalde Co., Harris Co., and Kerr Co.) and Ann Hendrickson (Edwards Co.). Ova acquired from the type locality in Kendall Co., were sent to Berry Nall in Falcon Heights, Texas (Rio Grande Valley), where they were reared on *Sideroxylon celastrinum*. The adult description of *L. rubritegula* is based on 20 pinned specimens; the larval description is based on two ex ova cohorts and one wild larva from the type locality. Forty-two genitalic slides of *Lactura* were examined: 29 on loan from the National Museum of Natural History (USNM) and 13 prepared by Tony Thomas for this study. Eight genitalic slides of *L. basistriga* and three of *L. rubritegula* were examined. Over the course of this study we examined the Nearctic *Lactura* holdings of the National Museum of Natural History (USNM) (Washington DC) (including primary types); Mississippi Entomological Museum (MEM) (Mississippi State, MS); Texas A&M University (TAMUIC) (College Station, TX), University of Connecticut (UCMS) (Storrs, CT), and the personal collections of Vernon A. Brou, Jr. (Albita Springs, LA); Edward C. Knudson (ECK) (Houston, TX); and James R. McDermott (JRM) (College Station, TX). CO1 barcodes for North American *Lactura* were compiled from holdings in the following institutions and personal collections: Canadian National Collection of Insects, Arachnids, and Nematodes, ECK, JRM, MEM, TAMUIC, UCMS, and USNM. We had access to 111 North American *Lactura* COI barcode submissions: for each of these we examined the associated voucher image. A neighbor-joining tree, using the default Kimura 2-P model, was generated by the Barcodes of Life Project (BOLD) (www.boldsystems.org) (Ratnasingham and Hebert 2007). DNA extraction, PCR amplification, and CO1 barcode sequencing were performed at the Canadian Centre for DNA Barcoding (Centre for Biodiversity Genomics – University of Guelph) using their standard protocol (Wilson 2012). Barcode sequences for the holotype and paratypes of *L. rubritegula* were deposited in GenBank. SimpleMappr (www.simplemappr.net) was used to generate the geographic distribution point map (Shorthouse 2010). Type material has been deposited in the USNM, TAMUIC, and UCMS.

## Taxonomy

### Lactura rubritegula

Taxon classificationAnimaliaLepidopteraLacturidae

Matson & Wagner sp. n.

http://zoobank.org/8AFB08C0-9E75-4DBC-BA69-6073EB35FA11

**Figures 1, 2.**
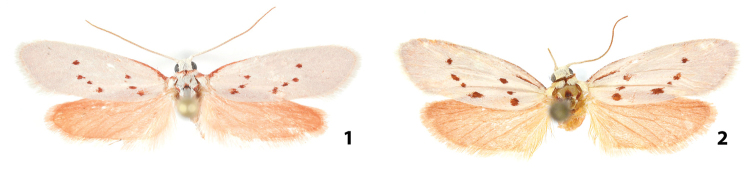
Adults of *Lactura rubritegula* and *L. basistriga*. **1** HOLOTYPE male *Lactura rubritegula*. TX: Kendall Co., Boerne, D. Cain Home (29°52'51"N, 98°36'51"W), 27 April 2015, David Wagner & Delmar Cain colls., CO1 Barcode DLW-000816 Genitalia slide # TAM-2017-002 **2** Female *Lactura basistriga*. TX: Cameron Co. Sabal Palm Grove (25°51'9"N, 97°25'3.8"W), BBN15#27.a, larva collected 25 April 2015; emerged 21 May 2015, Berry Nall coll., Host: *Sideroxylon celastrinum*, Genitalia slide # TAM-2017-005.

**Figures 3–6.**
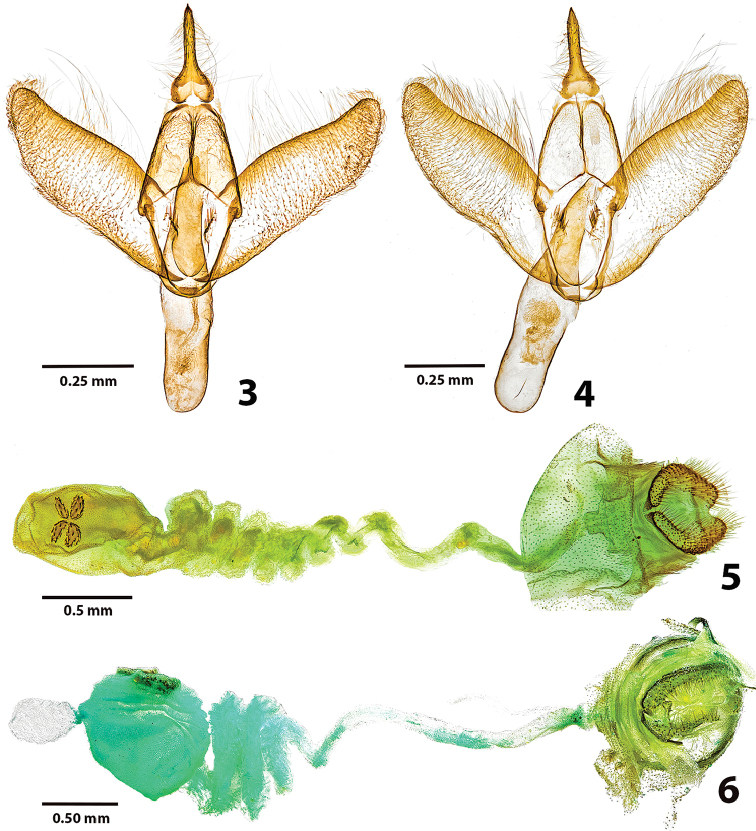
*Lactura* genitalia. **3** HOLOTYPE male *Lactura rubritegula* TX: Kendall Co., Boerne, D. Cain Home (29°52'51N, 98°36'51"W), 27 April 2015, David Wagner & Delmar Cain colls., CO1 Barcode DLW-000816 Genitalia slide # TAM-2017-002 **4** Male *Lactura basistriga* TX: Hidalgo Co., Bentsen St. Pk., 30 April 1995, Ed Knudson coll., Genitalia slide # TAM-2017-003, COI Barcode DLW-000513 **5** Female *Lactura rubritegula*. TX: Kendall Co., Boerne, D. Cain Home (29°52'51N, 98°36'50"W), 27 April 2015, David Wagner & Delmar Cain colls., Genitalia slide # TAM-2017-001 **6** Female *Lactura basistriga*. TX: Cameron Co. Sabal Palm Grove (25°51'9"N, 97°25'3.8"W), BBN15#27.a, larva collected 25 April 2015; emerged 21 May 2015, Berry Nall coll., Host: *Sideroxylon celastrinum*, Genitalia slide # TAM-2017-005.

**Figures 7, 8.**
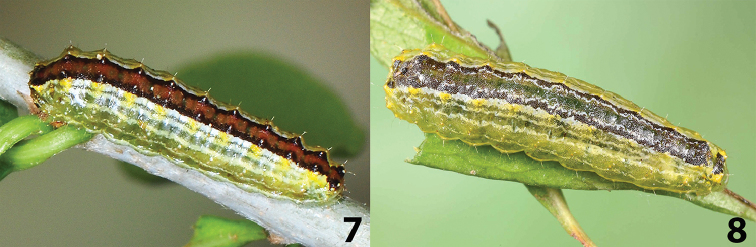
Larvae of *Lactura rubritegula* and *L. basistriga*. **7** Last instar ex ova *L. rubritegula*; mother: TX: Kendall Co., Boerne, 25 April 2015, David L. Wagner and Delmar Cain colls., DLW Lot: 2015D60, COI Barcode DLW-000568 **8** Last instar ex ova *L. basistriga*; mother: TX: Cameron Co., Southmost, Sabal Palm Audubon Sanctuary, 7 November 2013, David L. Wagner coll., DLW Lot: 2013L29, COI Barcode DLW-000466. Both clutches reared by Berry Nall on *Sideroxylon celastrinum*.

**Figure 9.**
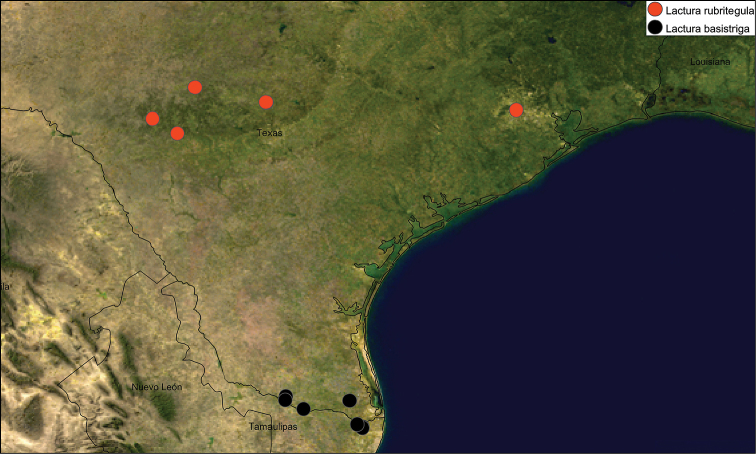
Geographic distribution of *Lactura rubritegula* (n=21, red) and *Lactura basistriga* (n=17, black). Single dots may represent >1 individuals.

**Figure 10.**
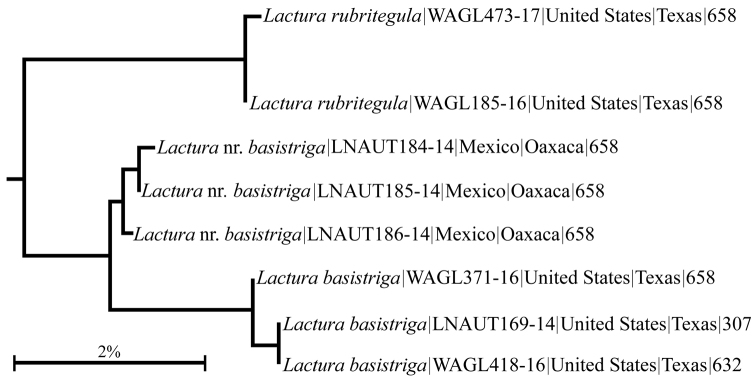
North American COI barcode tree for *Lactura basistriga* complex available as of 6 October 2017. Included for each taxon is the BOLD process ID, region, and sequence length.

Figs 1 , 3 , 5 , 7 , 9 , 10

#### Diagnosis.

*Lactura rubritegula* can be easily distinguished from its closest relative *L. basistriga* by the presence of red tegulae. It lacks the red subcostal dash that can be found in most forms of *L. basistriga* and the scattered flecking of red or brown scales characteristic of *L. subfervens*. Many, but not all, individuals can be distinguished by the basal displacement of the lowermost antemedial spot, somewhat enlarged upper postmedial spot, and the concave arcing (open to termen) of the three lower postmedial spots. We have not identified male genitalic characters that are unique to the new species. Females lack the small accessory pouch at the anterior end of the corpus bursae present in *L. basistriga* (Figs 5, 6) and some other *Lactura*; the anterior end of the ductus bursae is coiled six to seven times in *L. rubritegula*, but only 3–4 times in *L. basistriga*. Larvae are immediately distinguished from other Texas *Lactura* by their cinnamon-brown dorsum. In its various forms, the middorsal area of *L. basistriga* usually shows a green heart line. Supraspiracular stripes in *L. rubritegula* are about twice the width of those of *L. basistriga*. In the material that we have for study, the white pinacula are more prominent in *L. rubritegula* than those of other North American members of the genus.

#### Description.

***Adult male.*** Forewing length: 9.5–10.5 mm (n=12). Body salmon red. **Head.** Shiny, white decumbent scales over vertex and frons; lower frons sometimes with scattered pink scales. Labial palpus slightly porrect to straight, brick red, subequal to diameter of eye. Antenna filiform, 0.6 length of forewing; shiny, white decumbent scales over scape and basal 2/5^ths^, transitioning to admixture of white and red scales; distal 1/5^th^ brick red. **Thorax.** Predominantly white. Patagium and tegula red basally. Medial mesothoracic red spot flanked posterolaterally by ellipsoid red spots. Coxa and femora with whitish scaling along lower surface, other surfaces and segments brick red. Epiphysis well developed about ¼ protibial length. **Forewing.** Pearly white, with seven blood- to mahogany-red spots in oblique antemedial and postmedial series; without scattered dark scales (of *L. subfervens*). Antemedial row with three spots; lower spot usually displaced basally and often smaller than middle spot; postmedial series with four spots: uppermost usually larger or subequal to that below it; lower three forming straight line or (more commonly) slightly concave arc open to termen. (These same three spots often form convex arc in *L. basistriga* due to basal displacement of lowermost spot.) Basal red scaling along costa narrows and ends before antemedial spots. Underside red with white fringe scales. **Hindwing.** Uniformly light red, above and below, with elongate white fringe scales. **Abdomen.** Dorsum and sides brick red; venter white; paired ventral androconial brush composed of 20–40 white, thin scales hidden in intersegmental area between A5 and A6; second, subdorsal, paragenital androconial tuft of >40 straw-colored scales at base of tegumen. **Male genitalia.** Uncus basally cordiform; medial and distal part cylindrical, strongly down curved, ending in thorn-like spine. Tegumen lung shaped with strong medial crease. Valva elongated oval, 2.5× longer than wide, costa slightly concave pre-apically; broadly rounded; outer margin with shorter, thicker scales; lateral lobe of juxta with 6–8+ thickened spiniform setae. Vinculum narrow, U-shaped. Aedeagus exceeding length of valva; thickest at midlength; base broadly rounded; apex about half width of middle section, ending in short knob (Fig. 3). ***Female.* Forewing length.** 10–12 mm (n=9). Outwardly undifferentiated from male. **Female genitalia.** Papillae anales bowed inward, about 4× longer than wide, with long setae. Apophyses short and not especially well differentiated, approximately equal in length. Antrum thickened, hat shaped. Ductus bursae distally unmodified, anterior 2/3 spiraled into 6–7 tight whirls before entering corpus bursae; four strongly toothed signa arranged into two groups; corpus bursae ellipsoid, lacking anterior accessory pouch.

#### Description of living final instar.

Glossy pale green with broad cinnamon-brown middorsal stripe outwardly edged with black; white subdorsal and two, wavy-edged, pale supraspiracular stripes extending from T1–A8. Larger primary setae borne from minute white spots (~pinacula). Dorsum with black transverse lines at unions of each segment. White D1 pinacula from otherwise black warts. D2 seta also from white spot at apex of yellow wart with yellow washing down to SD seta. Thin, vague, wavy, pale spiracular stripe immediately ventral to light-orange spiracles, as well as single, white, straight-edged subventral stripe equal in width to supraspiracular stripes. Prothoracic shield well differentiated, medially divided, mostly black, although with little pigment deposition along its anterior and lateral margins (Fig. 7). Head black, partially retracted into prothorax.

#### Type material examined.

**Holotype male**, dry pinned (Figs 1, 3) TX: Kendall Co., Boerne, D. Cain Home (29°52'51"N, 98°36'51"W), 27 April 2015, David Wagner & Delmar Cain colls., CO1 Barcode DLW-000816 Genitalia slide # TAM-2017-002, Deposited at USNM, Washington D.C., USA. **Paratypes adults**. (10♂, 9♀): TX: Kendall Co., Boerne, D. Cain Home (29.8808°, -986139°), 26 April 2017 – 02 June 2017, Delmar Cain coll. (3♂, 4♀) (UCMS); TX: Kendall Co., Boerne, D. Cain Home (29°52'51N, 98°36'50"W), 27 April 2015, David Wagner & Delmar Cain colls., Genitalia slide # TAM-2017-004, TAM-2017-001, DLW-000568 (2♂, 2♀) (UCMS); TX: Kendall Co., Boerne, Clear Creek Circle (29°52'51N, 98°36'50"W), 27 April 2015, (ex ova; DLW Lot 2015D60) emerged 27 May 2016, David Wagner & Delmar Cain colls., reared on *Sideroxylon celastrinum*, CO1 Barcode DLW-000280 (1♂) (UCMS); TX: Edwards Co., 1.3 mi NW Camp Wood (29.6822°, -100.0289°), 23 April 2016, Ann Hendrickson coll., (1♀) (UCMS); TX: Edwards Co., 1.3 mi NW Camp Wood, (29.6822°, -100.0289°), 11–25 April 2017, Ann Hendrickson coll., CO1 Barcode DLW-000817 (3♂) (TAMUIC); TX: Uvalde Co., Concan, 23–27 April 2017, Ed Knudson coll., (1♂, 1♀) (USNM); TX: Kerr Co., 10 mi. W. of Hunt, 20 May 1995, Ed Knudson coll., (1♀) (UCMS).

#### Other material examined.

***Adults.*** TX: Harris Co., Spring Valley, 28 May 1985, leg. E. Knudson **Larvae.** TX: Kendall Co., Boerne, Clear Creek Circle (29.8807°, -98.6146°), 11 May 2017, Delmar Cain coll., beaten from *Sideroxylon lanuginosum* (n=1). TX: Kendall Co., Boerne, 29°52'51N, 98°36'50"W, ex ova from female 27 April 2015, DLW Lot: 2015D60, David Wagner & Delmar Cain colls., (n=6) (UCMS).

#### Distribution.

Hill Country around San Antonio, Texas, westward to Edwards and Uvalde counties, but range still unclarified due to taxonomic confusion with *L. basistriga* and other *Lactura*. A single specimen, seemingly out of range, was taken by Ed Knudson in Harris Co., Texas (Spring Valley). Range likely extends into Mexico.

#### Etymology.

The new species is named for the extensive red scaling through the basal half of the tegula, much of which is visible from above—red scales immediately distinguish both sexes from other members of the *Lactura basistriga* group that occur in the Rio Grande Valley and northern Mexico, although similar scaling occurs in *L. subfervens*.

#### Biology.

Larvae are specialists on *Sideroxylon* (formerly *Bumelia*) (Family Sapotaceae). *S. lanuginosum* is the only *Sideroxylon* that occurs at the three known localities for the species. Ex ova larvae of *L. rubritegula* were reared to maturity on *S. celastrinum* in captivity. Larvae preferred young leaves and rejected (and failed) on older leaves.

Peak flight of *L. rubritegula* appears to be tied to spring rains and the availability of new foliage. The moth begins flying in the second half of April. The majority of records are centered around the end of April and first half of May following the flights of *L. subfervens* and *L. pupula* at the type locality. It is unclear if early June captures represent late emergers or a small facultative second generation. An ex ova larva that was reared from a female taken on 25 April 2015 did not emerge until 27 May 2016.

In captivity the larvae typically feed from leaf undersides. Similar to other members of the genus, larvae emit slimy exudate from their integument; the function of the exudate remains unknown. At least in captivity, we often found feculae adhering to our caterpillars that remained attached until the next molt. Mature larvae spin a dense, red-brown cocoon in leaf litter or over soil. Presumably summer, fall, and winter months are passed as a prepupa.

## Discussion

So far as known, *Lactura rubritegula* has a curiously restricted range relative to that of its hostplant, *Sideroxylon lanuginosum* (gum bully)—the most widely distributed *Sideroxylon* in the United States. Gum bully’s range extends from Arizona to southern Kansas, Missouri, and Illinois, eastward to Georgia and Florida, and southward into Mexico. The moth is known principally from the southern Hill Country in the vicinity of San Antonio, westward and southward. Ed Knudson took a single specimen in west Houston (Spring Valley), which would suggest that the moth has simply been overlooked over parts of its range. We did not find additional examples in his collection, nor did we find any in the USNM collection, which includes Andre Blanchard’s extensive Texas collection.

We had access to barcode data for 111 North American *Lactura* specimens representing approximately 15 species-level taxa. The species cluster that includes *L. rubritegula* is shown in Fig. 10, henceforth we refer to this group as the *basitriga* complex. Barcode data for the group includes group includes *basistriga* (n=3; southern Texas), near basistriga (n=3; Oaxaca, Mexico), and the new species (n=2; Boerne, Texas). *L. rubritegula* shows an (uncorrected) divergence of 3.6% to its nearest neighbor in the *basistriga* group (from Oaxaca) and a 4.8% divergence from its nearest Texas neighbor, nominate *basistriga*.

Taxonomically, *Lactura* has proven itself to be a challenging genus to some of North America’s top systematic microlepidopterists, past and present, including August Busck, Harrison Dyar, and Ronald Hodges. Larval phenotypes and COI barcodes guided our understanding of this group by unmasking a tangle of misidentifications and segregating otherwise cryptic lineages. Based on our assessment of the North American fauna informed by larvae, life history data, and CO1 barcodes, the identifications in institutional collections, BOLD, and GenBank are riddled with misidentifications—e.g., 46% of the North American specimens of *Lactura* in BOLD are misidentified. Much of the confusion traces to concepts of *L. subfervens*, a highly variable taxon whose phenotypes overlap with those of *L. basistriga*, *L. rubritegula*, *L. psammitis* (Zeller, 1872) (TAM unpubl. data). Of the 52 specimens of *L. subfervens* and in BOLD, 65% of these appear to be misidentified.

We have attempted to rear two ex ova clutches of *Lactura rubritegula* from the type locality. Females captured on 25 April 2015 and 13 June 2017 were reared successfully by Berry Nall on *Sideroxylon celastrinum* in Falcon Heights, Texas. Pupae from the first cohort yielded an adult the following spring that is part of the paratype series. However, a wild larva, presumably an antepenultimate instar, found feeding on *S. lanuginosum* at the type locality in Boerne, Texas, on 13 June 2017 by Delmar Cain, straggled and died even when sleeved on the same branch from which it came, perhaps because the *Sideroxylon lanuginosum* foliage was too aged by mid-June to be acceptable. Whereas many Texas moths from arid and semi-arid habitats fly in almost any month when there has been appreciable rainfall, we only have late spring captures for *L. rubritegula*.

In contrast to the phenotypically uniform and confusing adults, the larvae of North American *Lactura* can be reliably identified to species. Larval features even unambiguously differentiate regional segregates within the two most widespread North American species, *L. pupula* and *L. subfervens*. Likewise barcodes disambiguate the North America species in that they align with our larval, life history, and biogeographic data. The confused nature of barcode data in BOLD appears to be entirely due to the misapplication of names by those making the initial submissions. We are currently collecting data for eight nuclear genes and preparing additional genitalic dissections for the North American fauna, and will await the outcome of these studies before making formal synonymies, producing adult and larval keys, and emending current BOLD determinations for the *Lactura* that occur north of Mexico.

Perhaps not surprisingly, for nearly every North American *Lactura* species there appears to be a closely related Mexican sister taxon, and in some case a species cluster in BOLD. There is much taxonomic work to be done, especially southward into Central and South America. We suspect the Nearctic fauna will continue to yield cryptic species and otherwise new species for decades, and that molecular data will play a critical role in getting this genus of look-a-likes properly partitioned into valid evolutionary units.

## Supplementary Material

XML Treatment for Lactura rubritegula
